# OryzaGP: rice gene and protein dataset for named-entity recognition

**DOI:** 10.5808/GI.2019.17.2.e17

**Published:** 2019-06-26

**Authors:** Pierre Larmande, Huy Do, Yue Wang

**Affiliations:** 1 UMR DIADE, Institute of Research for Sustainable Development (IRD), F-34394 Montpellier, France; 2 ICT Lab, University of Science and Technology of Hanoi (USTH), 100000 Hanoi, Vietnam; 3 Database Center for Life Science (DBCLS), Chiba 277-0871, Japan

**Keywords:** named-entity recognition, natural language processing, Oryza sativa, plant molecular biology, rice, text mining

## Abstract

Text mining has become an important research method in biology, with its original purpose to extract biological entities, such as genes, proteins and phenotypic traits, to extend knowledge from scientific papers. However, few thorough studies on text mining and application development, for plant molecular biology data, have been performed, especially for rice, resulting in a lack of datasets available to solve named-entity recognition tasks for this species. Since there are rare benchmarks available for rice, we faced various difficulties in exploiting advanced machine learning methods for accurate analysis of the rice literature. To evaluate several approaches to automatically extract information from gene/protein entities, we built a new dataset for rice as a benchmark. This dataset is composed of a set of titles and abstracts, extracted from scientific papers focusing on the rice species, and is downloaded from PubMed. During the 5th Biomedical Linked Annotation Hackathon, a portion of the dataset was uploaded to PubAnnotation for sharing. Our ultimate goal is to offer a shared task of rice gene/protein name recognition through the BioNLP Open Shared Tasks framework using the dataset, to facilitate an open comparison and evaluation of different approaches to the task.

**Availability:** A part of the OryzaGP dataset is publicly available through PubAnnotation (http://pubannotation.org/projects/OryzaGP). The full dataset will become available soon after a portion to be a hidden test data set is determined.

## Introduction

The last few decades have witnessed a massive explosion of information in the life sciences. However, an important proportion of this information, relevant to this field, is not available from databases, but is instead present in unstructured scientific documents, such as journal articles, reviews, abstracts, and reports. Agronomy is an overarching field that is comprised of diverse domains such as genetics, plant molecular biology, ecology and soil science [1]. Despite advancements in information technology, scientific advancements in agronomy are still commonly based on text. To effectively develop applications to improve crop production through sustainable methods, however, it is important to overlap research findings from these various subdomains, as they are highly interconnected. However, the collection of content is growing continuously, and the information currently available is unstructured text. Using these resources more efficiently, and taking advantage of associated cross-disciplinary research opportunities, poses a major challenge to both biologists and information technologists. One important subtask of information extraction is to identify biological entities, and their classifications, an endeavor known as named-entity recognition (NER).

Identifying biological entities, from text, is not trivial. Despite the existence of many available approaches to handle this problem in general, and in biomedical domains in particular, few comprehensive studies have been implemented for plants, especially rice. Moreover, we found that rare benchmarks are available for many plant species, but none for rice. Thus, taken together, we faced various difficulties to exploit advanced machine learning methods, for the accurate analysis of rice.

## Objective

On the large scale, we are currently building a Resource Description Framework (RDF) knowledge base termed Agronomics-Linked Data (AgroLD [2], http://www.agrold.org). This knowledge base is designed to integrate data from various public, plant-centric databases such as Gramene [3], Oryzabase [4], and TAIR [5], to name a few. The aim of the AgroLD project is to provide an integrated portal for both bioinformatics and domain experts, to exploit a homogenized data model for filling knowledge gaps. Using this landscape, we aim to extract relevant information from the literature, to enrich the content of integrated datasets.

Due to the scope of the project, we exploited information from the Oryzabase database to build a dataset aimed to recognize named text entities such as rice genes and proteins. Our main purpose was to solve NER of rice biological entities, to find the best approach. By sharing this dataset on the PubAnnotation platform and be available at the *BioNLP Open Shared Tasks* (BioNLP-OST, https://2019.bionlp-ost.org), we invited participants to implement their own methods to solve NER tasks for this dataset. Furthermore, to evaluate the performances, we compared their approaches, implemented during the task, with our method [6], implemented before the hackathon.

## Contribution

In this project, we used data from Oryzabase (http://pubannotation.org/projects/OryzaGP), a rice comprehensive database for Oryza sativa species published online since 2,000 by Japanese researchers. The latest version of Oryzabase contains 21,739 of rice genes, collected from 44,837 distinct scientific articles. Consequently, we used this information to create the basis of the OryzaGP dataset. Then we used PubMed as a resource to collect the raw data that was later preprocessed to compose the dataset, and developed a custom script implementing the BioPython library to query and retrieve the specific abstracts from PubMed. However, a number of scientific articles were not available in the PubMed database, due to some historical issues and lack of published resources. Due to the limited access of some resources, 10,400 articles were processed after filtering. The detailed raw data is shown in Table 1.

**Fig. 1.**
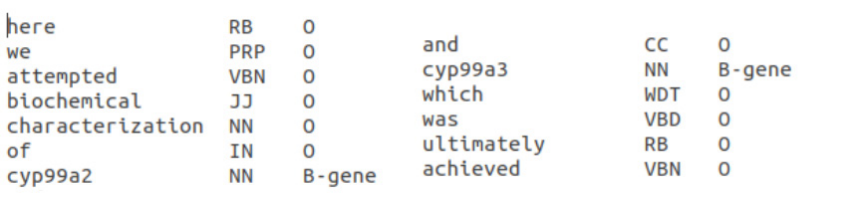
Example of data after pre-processing.

By focusing on the entities of the rice genome, we used the Oryzabase gene list as the ground truth to build up our dataset by keyword matching terms. The first step to preprocessing the data was filtering to remove special characters from the raw data. In fact, due to the number of articles used, the time range of articles was also wide, in that several articles were published in previous decades. To handle the problems of OCR-errors (which appear in the scanned text), we manually removed all the null and nonsense characters in utf-16 in the raw text. All the work was processed by our scripts and then after first step preprocessing. Moreover, we added part-of-speech (POS) tags for each word, to define its type with the aim, to ensure the accuracy of the identification of entities. The POS tagging process is supported by the Natural Language ToolKit (NLTK). To tokenize data, each word was considered a token, given in the following lines; one token per line, and included three tabs: the word itself, the POS tag, and the entity type (Fig. 1). To minimize the errors of inaccurate tags assignation when running the script, preprocessed data were checked manually, based on the existing resources (Oryzabase gene list, etc.)

During the 5th Biomedical Linked Annotation Hackathon (BLAH5, http://blah5.linkedannotation.org/), a portion of the dataset (29,098 annotation instances made to 6,107 abstracts) was uploaded to the PubAnnotation repository (http://pubannotation.org/projects/OryzaGP), which uses JSON (JavaScript Object Notation) as its default format, to store annotations. Resultantly, the dataset is accessible through or downloadable from PubAnnotation. Sharing it through PubAnnotation also means that the dataset can be compared to annotations from other projects if they share the same documents [7].

## Future Work

Our ultimate goal of sharing the dataset is to offer a shared task of rice gene/protein NER, through the BioNLP Open Shared Tasks (BioNLP-OST) framework, to facilitate open comparison and evaluation of various approaches to the task. Toward the goal, we will further upload remaining annotation data, while keeping some portion of it hidden for test dataset.

## Figures and Tables

**Table 1.** Description of the dataset

Name	OryzaGP
Text genre	Article
Text type	Abstract & title
Entity type	Gene, protein
No. of articles	10,400
No. of sentences	75,096
No. of words	2,697,726
